# Quantifying superparamagnetic signatures in nanoparticle magnetite: a generalized approach for physically meaningful statistics and synthesis diagnostics†

**DOI:** 10.1039/d3sc02113k

**Published:** 2023-06-15

**Authors:** Kyle M. Kirkpatrick, Benjamin H. Zhou, Philip C. Bunting, Jeffrey D. Rinehart

**Affiliations:** a Department of Chemistry and Biochemistry, University of California – San Diego La Jolla California 92093 USA jrinehart@ucsd.edu; b Materials Science and Engineering Program, University of California – San Diego La Jolla California 92093 USA

## Abstract

Magnetization is a common measurable for characterizing bulk, nanoscale, and molecular materials, which can be quantified to high precision as a function of an applied external field. These data provide detailed information about a material's electronic structure, phase purity, and impurities, though interpreting this data can be challenging due to many contributing factors. In sub-single-domain particles of a magnetic material, an inherently time-dependent rotation of the entire particle spin becomes possible. This phenomenon, known as superparamagnetism (SPM), simultaneously represents a very early size-dependent property to be considered, while being one of the least explored in the current quantum materials era. This discrepancy is, at least in part, due to the need for models with less built-in complexity that can facilitate the generation of comparative data. In this work, we map an extensive dataset of variable-size SPM Fe_3_O_4_ (magnetite) to an intrinsic statistical model for their field-dependence. By constraining the SPM behavior to a probabilistic model, the data are apportioned to several decorrelated sources. From this, there is strong evidence that standard measures such as saturation magnetization, *M*_S_, are poor comparative parameters, being dependent on experimental knowledge and measurement of the magnetic mass. In contrast, parameters of the intrinsic probability distribution, such as the maximum susceptibility, *χ*_max_, are far better suited to describe the SPM behavior itself and do not propagate unknown magnetic mass error. By confining the data fitting to intrinsic variables of the model distribution, scaling parameters, and linear contributions, we find greater value in magnetic data, ultimately aiding potential synthesis diagnostics and prediction of new properties and functionality.

## Abstract 2

The characteristic statistical response of superparamagnetic materials under magnetic stimuli provides quantitative insight into structure–function relationships, facilitating comparative data interpretation and paving the way for the design of superior functional materials.

## Graphical abstract

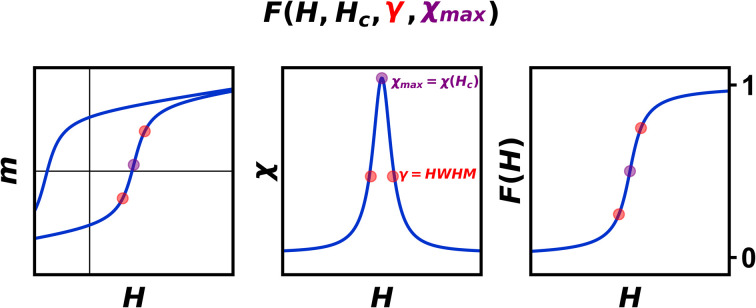

## Introduction

Much of nanoscience is predicated on the idea that fundamental properties of solid-state materials undergo radical changes when reduced to the nanoscale size regime, both as the result of the outsized role of the surface and from spatial confinement of the wavefunction. The enhanced and tunable functionality possible from such changes has resulted in a rapidly expanding array of synthetic and characterization techniques, as well as more intuitive and physically accurate models. In this pursuit, chemistry has played an increasingly important role in targeting and optimizing structure–property relationships on the nanoscale. One of the most intriguing areas to build structure–property insight is in nanoscale analogues of correlated magnetic materials. Such materials display fundamentally different behavior as a result of spatial confinement, enhancing the role of phonon coupling and giving rise to superparamagnetism (SPM) – a curious blending of permanent magnetism and paramagnetism. In SPM, the sub-single-domain confinement results in a collective angular momentum state with moment *M*_SPM_ = *M*_S_ cos(*θ*), where *θ* is the angle of rotation of the moment vector *θ* = [−*π*/2, *π*/2] with *θ* = ±*π*/2 representing the energy minima of a bistable double-well potential with spins totally aligned or anti-aligned. In this framework, the ground state is defined by an energetically isolated manifold of coupled spins known as a macro- or superspin. The macrospin has a collective spin that scales with the particle volume and takes on a time-dependent, high susceptibility switching behavior that is sensitive to a wide range of interactions and chemical modifications. Effectively, the SPM particle acts as a Curie paramagnet with a moment equivalent to the net moment of all contributions of the SPM nanoparticle, often 10^4^–10^5^*μ*_B_. The switchable nature of SPM finds applications at the intersections of many diverse fields^1^ such as biomedicine,^2^ electronics,^3,4^ sensing,^5^ imaging,^6^ rheology,^7^ and catalysis.^8^ With energy concerns driving the need for higher performance and materials scarcity driving efforts to diversify component resources, the need for understanding, control, and quantification of magnetic materials is extant.

Herein, we demonstrate how the stochastic nature of SPM can be leveraged in a general, physically meaningful model wherein the field-dependent magnetization curve is treated as a parameterized cumulative distribution function, *F*(*x*), of a Cauchy distribution (also known as the Lorentz or Cauchy–Lorentz distribution). Within this framework, we can decorrelate the intrinsic distribution of particle spin alignments from overall scaling and background paramagnetism. This method is designed to generate a consistent parameter space across samples displaying SPM to extract consistent, intuitive, and quantitative information from samples collected under a variety of conditions and with various imperfections. In essence, it acts as a pre-processing step to categorize parameters by their origin and constraints, dramatically reducing the noise in any further modeling based on specific quantum mechanical models. In a useful example, we demonstrate how peakshape parameters such as the maximum susceptibility, *χ*_max_, display a consistent linear trend with magnetic particle size while scaling parameters such as saturation magnetization, *M*_S_ do not.

**Fig. 1:**
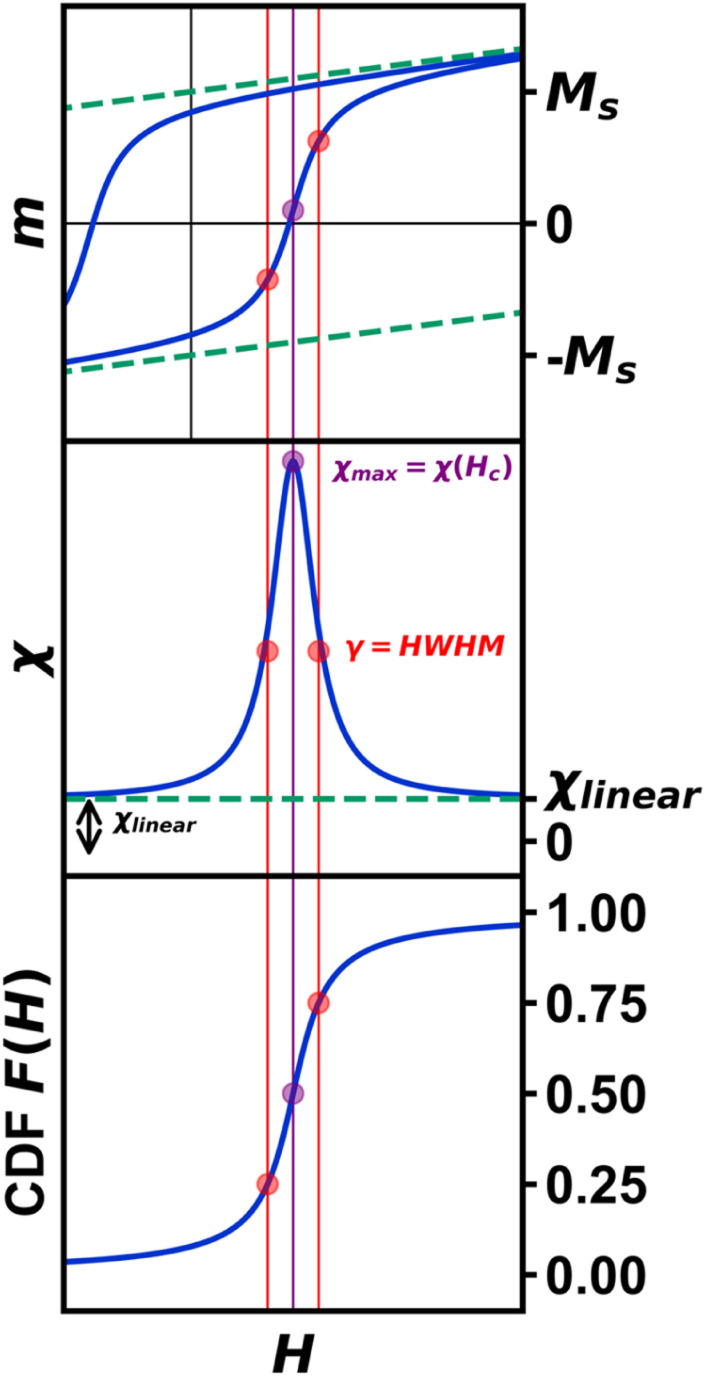
Magnetization curves (top), differential magnetization (*χ*) curve (middle), and corresponding Cauchy CDF (bottom). The effect of *χ*_linear_ (green dashed line) is demonstrated in the magnetization curve, as it obscures both the true saturation magnetization (*M*_s_) and coercive field (*H*_c_), however, it is depicted more clearly as the vertical offset of the differential magnetization curve at *H* = ±*∞*. Stemming purely from the statistical model, *γ* is represented in red as the half-width at half-max (HWHM). The susceptibility at *H*_c_, denoted *χ*_max_, is represented in purple.

Arguably the most common and information-rich characterization method of bulk, nanoscale, and molecular magnetic materials is the measurement of magnetization as a function of an external magnetic field (Fig. 1 and S1†). In nanomaterials, specific models have been employed to interpret magnetic moment *vs.* magnetic field in terms of structure^9,10^ and degree of crystallinity,^11–13^ as well as nanoscale-specific properties such as size,^14,15^ shape,^16,17^ and surface.^18,19^

In many cases, the correlation of properties with fit parameters depends strongly on the specifics of the material and sample form, making broad comparisons across many samples difficult. At the heart of the difficulty in applying generalizable models to nanoscale magnetism, especially colloidal nanoparticles, is the meaningful treatment of rapidly fluctuating forces arising from non-uniform physical systems (*i.e.*, particle distributions). Modeling complex data as a combination of both deterministic and stochastic forces is often achieved *via* solutions to stochastic differential equations. For example, the effect of particle size dispersity can be modeled through a lognormal distributed function.^20–28^ The most common solution to a Langevin equation used in magnetism corresponds to the macrospin limit 
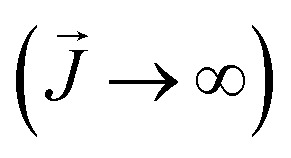
 of the Brillouin equation for saturation behavior of a discrete magnetic angular momentum vector, 
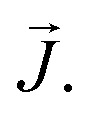
 The macrospin approximation of the SPM, however, means that the moment *vs.* external field behavior is better estimated as the net sum of the moment-weighted populations of fully aligned and anti-aligned states. From this standpoint, a magnetic field sweep can be re-normalized in the form of a cumulative distribution function (CDF; *F*(*H*)). This CDF describes a large population of macrospins aligned in one field direction changing their equilibrium population as field is swept until the equilibrium lies fully in the other direction. Due to the equilibrium nature of the system, the CDF endpoints of 0 and 1 are only reached at fields of *H* = ±∞. The use of such a model for data modeling and comparative analysis is of interest because any parameter distribution associated with it can be assigned to the intrinsic SPM behavior and is subject to the constraints of the distribution.

The Cauchy distribution (Fig. S2†) is a continuous, stable probability distribution that conforms to the requirements detailed above. Its cumulative distribution function (Fig. 1, eqn (1)) and probability density function (PDF; eqn (2)) are modified by the location parameter, *x*_0_, and the scale parameter, *γ*.^29^ As a stable distribution, the Cauchy distribution has applications in chemistry and physics,^30–32^ geology,^33^ engineering,^34^ and economics.^35^1
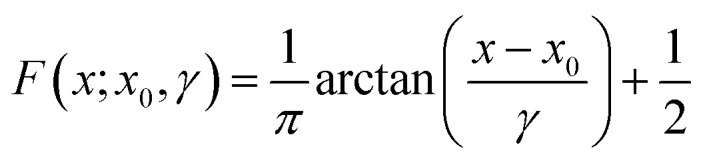
2
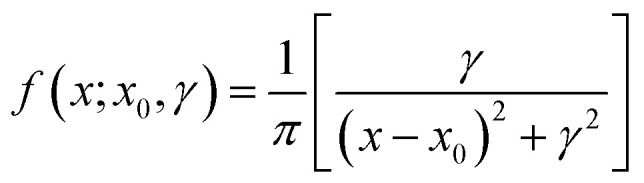
3
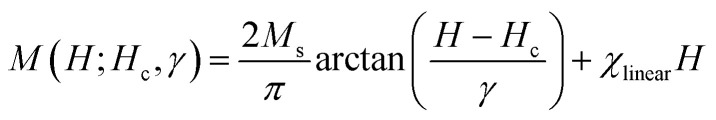


All data reported herein is fit to the CDF of an underlying Cauchy distribution that is scaled proportional to *M*_s_ with an additive parameter *χ*_linear_*H*, where *χ*_linear_ is a susceptibility term accounting for the sum response from paramagnetic and diamagnetic components. To extract consistent and quantitative information, the Cauchy distribution model is presented to provide insight into intrinsic SPM behavior. This model provides a material-independent formalism for standardized data collection and comparison, as well as a method for pre-processing data for validation and further analysis *via* deterministic models.

## Results and discussion

**Fig. 2:**
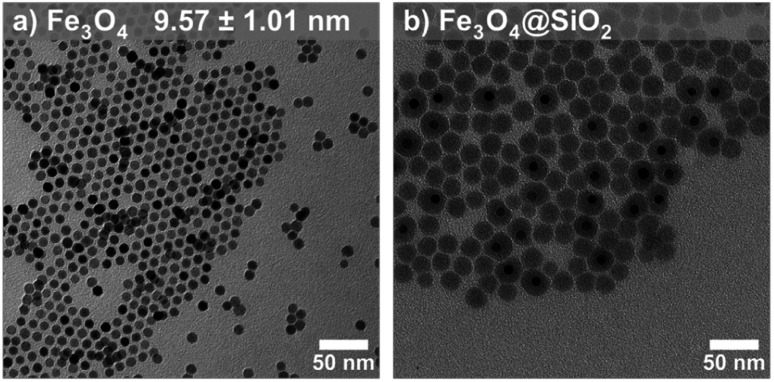
TEM images (a) 9.57 nm Fe_3_O_4_ nanoparticles and (b) Fe_3_O_4_@SiO_2_ nanoparticles.

Magnetite (Fe_3_O_4_) nanoparticles have a high magnetization and superparamagnetic susceptibility over a wide size range, making them desirable targets for magnetic optimization towards numerous applications.^36^ Magnetite nanoparticle samples of varying size (*d* = 5–12 nm) were synthesized according to our previously described method (Fig. 2a, S3a–e and Table S1†).^15^ The percent relative standard deviation (%RSD) among all sample diameters exhibits a notable degree of consistency, falling between 9.88% and 12.97%. Briefly, a stoichiometric powder form of iron oleate was used in the high temperature decomposition of iron oleate with octadecene and oleic acid as the solvent and surfactant, respectively. To avoid the commonly observed issue of overreduction of magnetite (Fe_3_O_4_) to wüstite (FeO), 5% O_2_ was flowed through the reaction during the reflux stage. One particularly difficult aspect of modeling SPM nanoparticle magnetization is the detection and quantification of interparticle interactions. To probe the effect of interparticle interactions, silica shells were grown onto each set of magnetite nanoparticles (Fig. 2b) through a previously described reverse microemulsion procedure.^37^ Briefly, silica shells were grown onto Fe_3_O_4_ by the hydrolysis of tetraethylorthosilicate (TEOS) in microemulsions of aqueous ammonia in cyclohexane. Empty silica shells further prevent interparticle interactions. The average diameter was determined *via* TEM. Magnetite phase purity was confirmed by pXRD (Fig. S4†). Finally, to account for variability in surface ligand mass by sample, Thermogravimetric Analysis (TGA) was carried out from 30 °C to 1000 °C under air (Fig. S5, S6 and Table S2†).

**Fig. 3:**
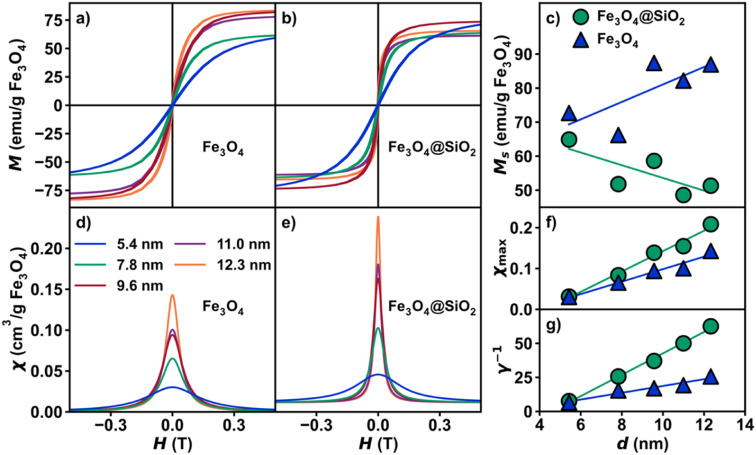
Magnetic properties. Plots of isothermal magnetization *vs.* magnetic field for (a) Fe_3_O_4_ and (b) Fe_3_O_4_@SiO_2_ at 300 K. (c) Plot of saturation magnetization *vs.* nanoparticle diameter (nm). Plots of derivative to fit (magnetic susceptibility) *vs.* magnetic field for (d) Fe_3_O_4_ and (e) Fe_3_O_4_@SiO_2_ at 300 K. (f) Plots of *χ*_max_*vs.* nanoparticle diameter (nm) and (g) *γ*^−1^*vs.* nanoparticle diameter (nm).

Magnetization *vs.* magnetic field studies (Fig. 3a and b) were conducted on all Fe_3_O_4_ and Fe_3_O_4_@SiO_2_ samples (*H* = −7 to 7 T; *T* = 300 K). A calibration of the magnetic field was carried out using a palladium standard with a precisely known susceptibility. This step is necessary for precise measurement of the low-field magnetization, as remnant fields of approximately ±30 Oe in the superconducting magnet can fluctuate over time. This small residual field leads to a difference between the recorded and true fields, resulting in an “inverted” hysteresis loop and false coercivities (details in ESI†). An interpolation was performed on the forward sweep of each curve, generating evenly spaced points to prevent biasing, then fit to eqn (3) for further analysis (Table S3†). The derivative, d*M*/d*H* of the fit function from each data set (Fig. 3d and e) represents the instantaneous magnetic susceptibility as a function of magnetic field. Visualizing d*M*/d*H* (proportional to the Cauchy PDF and also known as a Lorentzian lineshape) can be advantageous for various applications, such as Magnetic Particle Imaging (MPI),^38^ and provides intuition about parameters typically neglected in SPM analysis. These parameters include the maximum superparamagnetic susceptibility, *χ*_max_ (Fig. 3f), *γ* (Fig. 3g), and *χ*_linear_, the *y*-axis offset of d*M*/d*H vs. H* at *H* = ±∞ (Fig. 1). While some amount of *χ*_linear_ is common due to decoupled spins at the particle surface,^39^ from defects, or from molecular impurities, these effects are typically only observable at fields where the SPM macrospin is fully saturated.

One of the most interesting points to emerge from our quantification of SPM magnetization data by the Cauchy distribution function was the lack of conformity of the saturation magnetization, *M*_S_ to a distinct size trend. The size of the macrospin is expected to increase linearly with particle volume in the SPM regime, and we observe only a very weak trend (Fig. 3c). In a general sense, an increase in saturation magnetization with nanoparticle diameter is observed for Fe_3_O_4_ nanoparticles, while a slight decrease in saturation magnetization with diameter is observed in Fe_3_O_4_@SiO_2_. This difference in behavior could be attributed to surface effects at the interface between the two materials, although due to the effect of sample mass error, it is important to exercise caution when considering saturation magnetization. The difference in error of the sample mass is roughly 10^2^ times larger than the error in magnetic moment. To demonstrate the effect of error, 1000 samples following randomized normal distributions in magnetic moment and mass with corresponding typical errors were generated and plotted as a histogram of magnetic moment per gram (Fig. S7†). For these reasons, parameters based on susceptibility (*γ*, *γ*^−1^, *χ*_max_, *χ*_linear_) can supplement saturation magnetization to better describe the overall magnetic properties of SPM nanoparticles. The use of susceptibility-based terms is demonstrated in Fig. 3f and g, as both *χ*_max_ and *γ*^−1^ exhibit a strong linear dependence on nanoparticle diameter. By comparison, only a weak trend is evident in *M*_s_*vs. d* in (Fig. 3c), highlighting that *γ* is determined from the distribution function only and thus decorrelated from the scaling of the magnetization curve.

The effect of interparticle interactions *via* dipolar coupling is commonly neglected in measurements of nanoparticle SPM due to assumptions about its relative strength or how the behavior should manifest. It has been shown that the introduction of large silica shells can drastically reduce interparticle interactions compared to other methods. Indeed, from our ZFC-FC measurement, stark differences are observed between interacting *vs.* non-interacting samples (Fig. S8†). While non-interacting Fe_3_O_4_ nanoparticles are expected to exhibit a rise in the FC curve with decreasing temperature below the blocking temperature, as dipolar coupling strength increases, Fe_3_O_4_ nanoparticles (*e.g.*, randomly-close-packed powder assemblies) will exhibit a slight dip in the FC curve below the blocking temperature.^40,41^ Additionally, the non-interacting case results in a sharper ZFC curve. All five Fe_3_O_4_@SiO_2_ lack signatures of interparticle interactions, confirmed by the rise in the FC curves below the blocking temperature and a significant enhancement in peak sharpness (Fig. S9b†). To compare both nanoparticle sets, the Fe_3_O_4_ mass percentage in Fe_3_O_4_@SiO_2_ was determined by EDX (Table S2†). In applications such as MPI and granular magnetoresistance, the sharpness of the magnetization curve directly correlates with performance. In the absence of dipolar coupling, *χ*_max_ and *γ*^−1^ increase across all sizes, indicating a sharpening of the d*M*/d*H* peak. For example, the 12.3 nm sample exhibits an increase in *χ*_max_ of 106% between Fe_3_O_4_ and Fe_3_O_4_@SiO_2_.

**Fig. 4:**
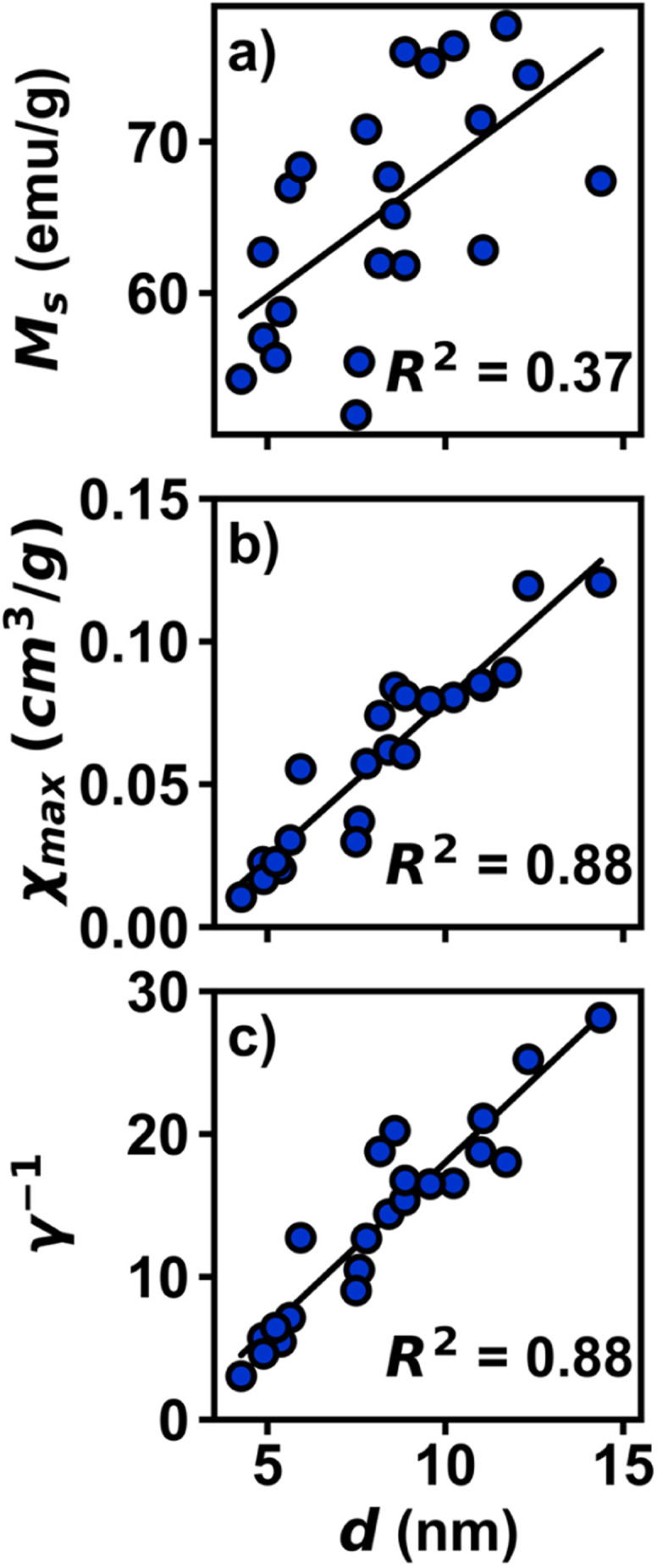
Plots of magnetic parameters for large dataset of Fe_3_O_4_ nanoparticles, plotted in per g of sample. (a) Plot of *M*_s_*vs. d*. (b) Plot of *χ*_max_*vs. d*. (c) Plot of *γ*^−1^*vs. d*.

As a test of the trends observed with *χ*_max_ and *γ*^−1^, an analysis across a larger dataset was performed (Fig. 4, Table S4†). An identical analysis was conducted using 22 Fe_3_O_4_ samples, synthesized over a period of two years with variations in iron oleate synthetic methods, and with diameters ranging from 4 nm to 14 nm. Each 300 K magnetization curve was fit to eqn (3). In a similar fashion to the smaller dataset, the larger dataset also exhibits a weak dependence of *M*_s_ on diameter (*R*^2^ = 0.37) with a stronger relationship of *χ*_max_ (*R*^2^ = 0.88) and *γ*^−1^ (*R*^2^ = 0.88) *vs.* diameter, thus demonstrating the viability of this method. The collection, analysis, and statistical modelling of large datasets holds the potential to uncover previously unobserved trends in magnetic nanoparticles and help identify impurities, phase mixtures, multi-domain relaxation effects, and other synthetic challenges often obscured by qualitative or overly parameterized models.

Following the strong trend of maximum magnetic susceptibility as a function of diameter, the ability to predict and target specific magnetic properties can become trivial. This may prove useful in biological and sensing applications requiring maximal magnetic susceptibility response within a targeted field range. For example, in MPI, the *γ* parameter (HWFM of *χ vs. d*) is indicative of spatial resolution.^38^ Another example is in nanocomposite magnetoresistance, where predicting the structure–property relationship correlates with magnetoresistance percentage and curve shape.^4^

As previously discussed, superparamagnetic nanoparticles are typically characterized with their native long-chain ligands present and thus contain a significant percentage (10–25% w/w) of diamagnetic organic material. The presence of diamagnetic material is wholly accounted for by the linear susceptibility term, *χ*_linear_. Therefore, any diamagnetic contribution can be effectively subtracted out, leaving just the contribution from the SPM portion. This concept is also observed in the Fe_3_O_4_@SiO_2_ samples, as the diamagnetic contribution from silica is entirely accounted for with *χ*_linear_.

**Fig. 5:**
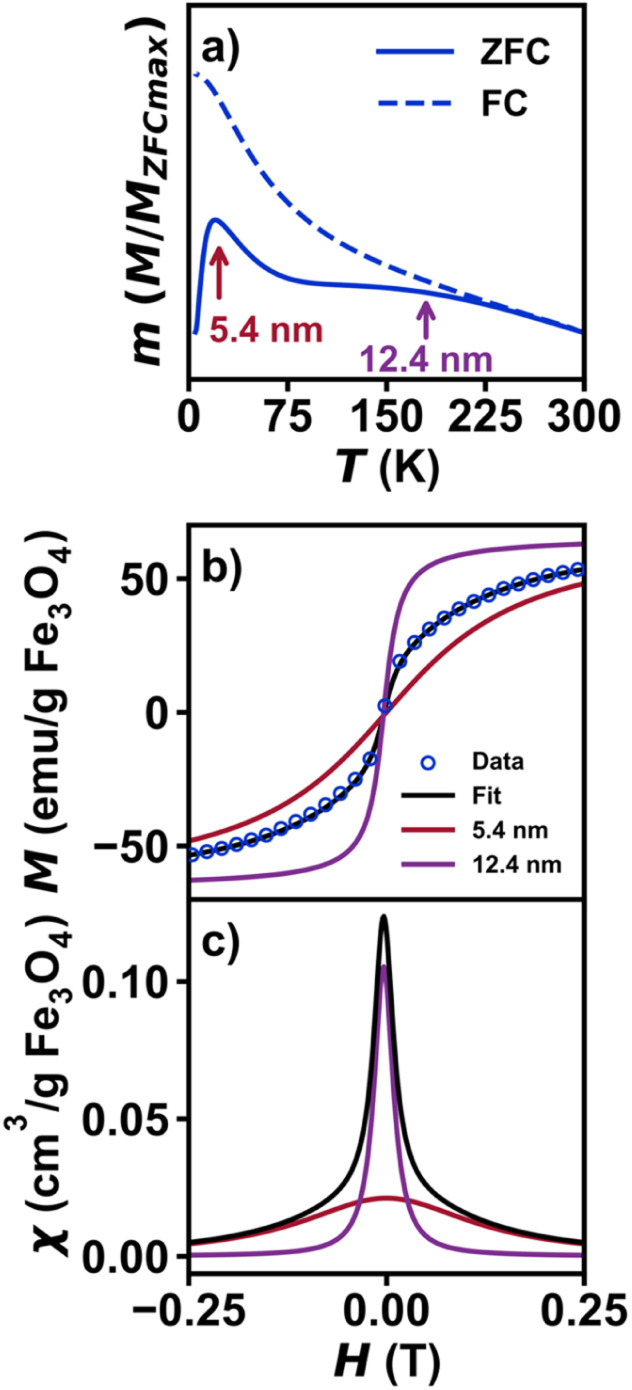
Magnetic data for a physical mixture of 5.4 nm Fe_3_O_4_@SiO_2_ and 12.3 nm Fe_3_O_4_@SiO_2_. (a) Plot of normalized zero-field cooled magnetization *vs.* temperature from 5–300 K under an applied field of 0.01 T. (b) Plot of isothermal magnetization *vs.* magnetic field for the physical mixture. The fit is shown in black, with contributions from 5.4 nm and 12.3 nm shown in red and purple, respectively. The fit was carried out using a sum of two unique Cauchy functions, with a relative ratio between the two, *p*, as an additional fit parameter. The *γ* and *H*_c_ parameters from the individual samples (Table S3†) were held constant, while *M*_s_ and *χ*_linear_ were allowed to vary to account for mass errors. (c) Plot of magnetic susceptibility *vs.* magnetic field, with contributions from the 5.4 nm and 12.3 nm shown in blue and orange, respectively. And (e) Fe_3_O_4_@SiO_2_ at 300 K. (f) Plots of *χ*_max_*vs.* nanoparticle diameter and *γ*^−1^*vs.* nanoparticle diameter.

Extending this concept to multiple independent SPM signals, the Cauchy method can deconvolute contributions from magnetic materials beyond the simple case of neat oleate bound and silica shelled iron oxide nanoparticles. While any sum of Cauchy distributions within an isolated particle will form a single Cauchy distribution (*e.g.*, representing a more complex energy manifold), a separate population of isolated magnetic components with different properties will be represented with a unique Cauchy function. To show this experimentally, a physical mixture was made with 5.4 nm Fe_3_O_4_@SiO_2_ and 12.3 nm Fe_3_O_4_@SiO_2_ to simulate a bimodal distribution that can result from colloidal synthesis. Contributions from each sample are observed in the ZFC-FC measurement (Fig. 5a). The magnetization curve was fit to a linear combination of two unique Cauchy functions (Fig. 5b) modified by a term, *p*, to account for the relative ratio of each component. The plot of magnetic susceptibility *vs.* magnetic field (Fig. 5c) of the mixture demonstrates that two unique distributions are necessary to fully describe the magnetization curve, while a single Cauchy CDF is a poor fit (Fig. S10†). A test for multiple distribution fits to interpret magnetization data may prove useful in applications beyond the SPM nanoparticles studied in this work. At minimum, it can help to prevent misinterpretation of mixed samples where qualitative interpretation of the data may result in poor conclusions and perpetuate synthetic difficulties.

## Conclusions

Statistical modelling is shown to improve the quantification of experimentally complex data of SPM nanoparticles. A generalized curve-fitting model is demonstrated based on the Cauchy distribution to better describe the overall magnetization curve. The preservation of underlying statistics is imperative for making accurate comparisons between datasets, with the potential to discover stronger trends, built upon new models, ultimately aiding material design.

## Data availability

All data underlying the findings of this study, along with any associated in-house code for processing and computational modeling, have been deposited in a structured and version-controlled repository on Zenodo. The dataset and codebase are openly available under the MIT License to foster transparency and reproducibility. These data can be accessed *via* the following DOI: 10.5281/zenodo.7987572.

## Author contributions

K. M. K. synthesized and characterized all materials and performed the fitting analyses. B. H. Z., P. C. B., and J. D. R. provided valuable insight into the synthetic methods and contributed to the data analysis. All authors contributed to the writing and editing of the manuscript.

## Conflicts of interest

There are no conflicts to declare.

## Supplementary Material

SC-014-D3SC02113K-s001
