# Supplementary material for: Quantifying superparamagnetic signatures in nanoparticle magnetite: a generalized approach for physically meaningful statistics and synthesis diagnostics
Source: Chem Sci. 2023 Jun 15;14(27):7589–94. doi: 10.1039/d3sc02113k (PMC10337765; doi:10.1039/d3sc02113k)
Supplement: SC-014-D3SC02113K-s001 [file SC-014-D3SC02113K-s001.pdf]

Electronic Supplementary Material (ESI) for Chemical Science.
This journal is © The Royal Society of Chemistry 2023

Supporting Information

Quantifying superparamagnetic signatures in nanoparticle magnetite: A generalized approach for physically meaningful statistics and synthesis diagnostics

Kyle M. Kirkpatrick[a], Benjamin H. Zhou[b], Philip C. Bunting[a], and Jeffrey D. Rinehart[*,a,b]

[a]Department of Chemistry and Biochemistry and [b]Materials Science and Engineering Program, University of California – San Diego, La Jolla, California 92093, United States

EXPERIMENTAL:

Materials

The reagents used were iron (III) chloride hexahydrate (97% Alfa Aesar), iron (II) chloride tetrahydrate (97% Fisher), sodium oleate (97% TCI), oleic acid (90%, Alfa Aesar) and 1-octadecene (90% Sigma Aldrich). ACS grade hexane, ethanol, and methanol were purchased from Fisher. Oleic acid was degassed and stored under vacuum in a Schlenk flask covered with aluminum foil. All other chemicals were used as received.

Synthesis of iron oleate

Iron oleate in its powder form was synthesized according to a previous literature procedure.[1]

Nanoparticle synthesis from iron oleate (FeOl)

Fe_3O_4 nanoparticles were synthesized and purified according to a previous literature procedure.[1]

Synthesis of Fe_3O_4@SiO_2 nanoparticles

Silica shelled iron oxide nanoparticles were synthesized in a reverse microemulsion method according to a literature procedure.[2] In a typical synthesis, 17 mL Igepal CO-520 was stirred in a 20 mL scintillation vial for two minutes, followed by the addition of 0.8 mg Fe_3O_4 nanoparticles in hexanes (1-10 mg/mL Fe_3O_4 dispersion in hexanes). Next, 0.13 mL NH_4OH was added dropwise, and the solution was stirred for five minutes, followed by the addition of 0.15 mL tetraethylorthosilicate (TEOS). The reaction was capped and allowed to stir at room temperature for 72 hours. The final purification step consists of a of an initial flocculation with methanol, centrifugation (7 minutes at 8500 rpm), redispersion in ethanol, flocculation with hexanes, and a final centrifugation step (7 minutes at 8500 rpm) to recover the Fe_3O_4@SiO_2 nanoparticles.

Characterization

Transmission electron microscopy was carried out using a FEI Spirit TEM operating at 120 kV, with images collected by a 2k x 2k Gatan CCD camera. TEM samples were prepared by drop-casting and air drying a dilute solution of nanoparticles in hexanes onto a carbon-coated copper TEM grid. Particles were analyzed in ImageJ using the default thresholding algorithm of sample sizes exceeding 1000 particles for all syntheses.

Powder X-ray diffraction was performed with a Bruker D8 Advance diffractometer using a Bruker Apex II Ultra CCD using Mo Kα (λ = 0.71073 Å) radiation.

Thermogravimetric analysis was carried out using a SDT650 instrument. Samples (10-20 mg) were loaded into a 90 μL alumina pan and heated under air from 30 °C to 1000 °C. The conversion of Fe_3O_4 to Fe_2O_3 was accounted for and confirmed via powder X-ray diffraction.

Energy dispersive X-ray analysis was performed on a Zeiss Sigma 500 SEM instrument operating at 15 kV. Samples were prepared by dropping powdered Fe_3O_4@SiO_2 samples onto conductive carbon tape. Each sample was analyzed at three locations on the stub for a minimum of 200 seconds each.

Magnetic measurements were carried out using a Quantum Design MPMS3 SQUID magnetometer. Nanoparticle samples were dried to a fine powder (1-2 mg), loaded into a VSM sample holder, and secured in a plastic straw. A calibration of the magnetic field was carried out using the palladium standard supplied by Quantum Design with a precisely known susceptibility, a necessary step to remove small remnant fields in the superconductive magnet. This small residual field leads to a difference between the recorded and true fields, resulting in an “inverted” hysteresis loop and an incorrect coercivity. Further details for this calibration are found in Quantum Design Application Notes.[3,4]

Fitting Analysis

Fitting of the magnetization curves were performed in Python. To remove emphasis on any particular portion of the magnetization curve, a linear interpolation was performed on the forward sweep of the magnetization curve, generating 10,000 equally spaced points. Fitting was carried out using the default least squares method of the LMFIT package according to the initial values and constraints below. The data and code for this analysis can be found at DOI: 10.5281/zenodo.7987572.

Parameter	Initial Value	Min	Max
H_c (Oe)	0	-100	100
γ	0.01	1×10^{-5}	1
χ_{linear} (cm^3/g Fe_3O_4)	0	-2	2
M_s (emu/g Fe_3O_4)	75	1	100

Table S1. Summary of synthesis parameters for Fe_3O_4 nanoparticles.

Size (nm)	Stdev	FeOl (g)	OA (g)	ODE (g)
5.43	0.62	0.50	0.78	6.40
7.83	1.05	0.25	0.29	7.10
9.57	1.01	1.00	0.77	5.87
11.00	1.08	1.00	1.16	6.48
12.33	1.61	0.50	0.39	6.78

Table S2. Iron oxide percentages for Fe_3O_4 and Fe_3O_4@SiO_2 nanoparticles.

	Fe_3O_4 (native oleate ligands)	Fe_3O_4@SiO_2
**Size (nm)**	**Fe_3O_4 % (w/w)**	**Fe_3O_4 % (w/w)**
5.43	74.862	8.742
7.83	79.733	2.315
9.57	86.115	3.996
11.00	86.920	2.618
12.33	85.611	5.357

Table S3. Magnetic hysteresis loop fit parameters to Cauchy distribution.

Fe_3O_4						
**Size (nm)**	**M_s (emu/g Fe_3O_4)**	**H_c (Oe)**	**γ**	**χ_{linear} (cm^3/g Fe_3O_4)**	**χ_{max} (cm^3/g Fe_3O_4)**	**χ^2 of fit**
5.43	72.69	0.40	0.154	-3.77×10^{-6}	0.030	941.5
7.83	66.22	0.80	0.065	-7.34×10^{-6}	0.065	1562.7
9.57	87.38	1.23	0.059	-1.27×10^{-5}	0.094	5247.7
11.00	82.21	0.91	0.052	-1.40×10^{-5}	0.101	3894.1
12.33	86.95	1.76	0.039	-8.05×10^{-6}	0.143	2051.2
$Fe_3O_4@SiO_2$						
**Size (nm)**	**M_s (emu/g Fe_3O_4)**	**H_c (Oe)**	**γ**	**χ_{linear} (cm^3/g Fe_3O_4)**	**χ_{max} (cm^3/g Fe_3O_4)**	**χ^2 of fit**
5.43	64.89	-0.08	0.132	-2.89×10^{-5}	0.031	222.1
7.83	51.79	-0.07	0.039	-1.48×10^{-4}	0.083	27.3
9.57	58.60	0.36	0.027	-1.05×10^{-4}	0.139	32.5
11.00	48.58	0.47	0.020	-1.29×10^{-4}	0.155	120.9
12.33	51.32	0.80	0.016	-6.35×10^{-5}	0.208	317.3

Table S4. Magnetic hysteresis loop fit parameters to Cauchy distribution for large dataset.

Size (nm)	M_s (emu/g Fe_3O_4)	γ	χ_{linear} (cm^3/g Fe_3O_4)	χ_{max} (cm^3/g Fe_3O_4)
4.26	54.33	0.329	9.17×10^{-7}	0.011
4.87	62.73	0.175	8.93×10^{-6}	0.023
4.89	57.04	0.217	-1.58×10^{-5}	0.017
5.23	55.73	0.155	-3.36×10^{-5}	0.023
5.38	58.77	0.183	1.32×10^{-5}	0.020
5.64	66.96	0.140	-3.72×10^{-7}	0.030
5.93	68.33	0.079	-5.56×10^{-6}	0.055
7.49	51.94	0.111	5.10×10^{-6}	0.030
7.58	55.46	0.095	3.00×10^{-6}	0.037
7.78	70.83	0.079	-7.43×10^{-6}	0.057
8.16	61.97	0.053	-1.29×10^{-6}	0.074
8.41	67.69	0.069	-4.83×10^{-6}	0.062
8.58	65.23	0.049	-8.82×10^{-6}	0.084
8.86	61.83	0.065	4.88×10^{-5}	0.061
8.87	75.94	0.060	-2.44×10^{-6}	0.081
9.57	75.22	0.061	-1.02×10^{-5}	0.079
10.23	76.33	0.060	-1.03×10^{-5}	0.080
10.99	71.44	0.053	-1.15×10^{-5}	0.085
11.06	62.84	0.047	-6.87×10^{-6}	0.084
11.71	77.67	0.055	-1.26×10^{-6}	0.089
12.33	74.42	0.040	-6.39×10^{-6}	0.119
14.37	67.40	0.036	-3.40×10^{-6}	0.121

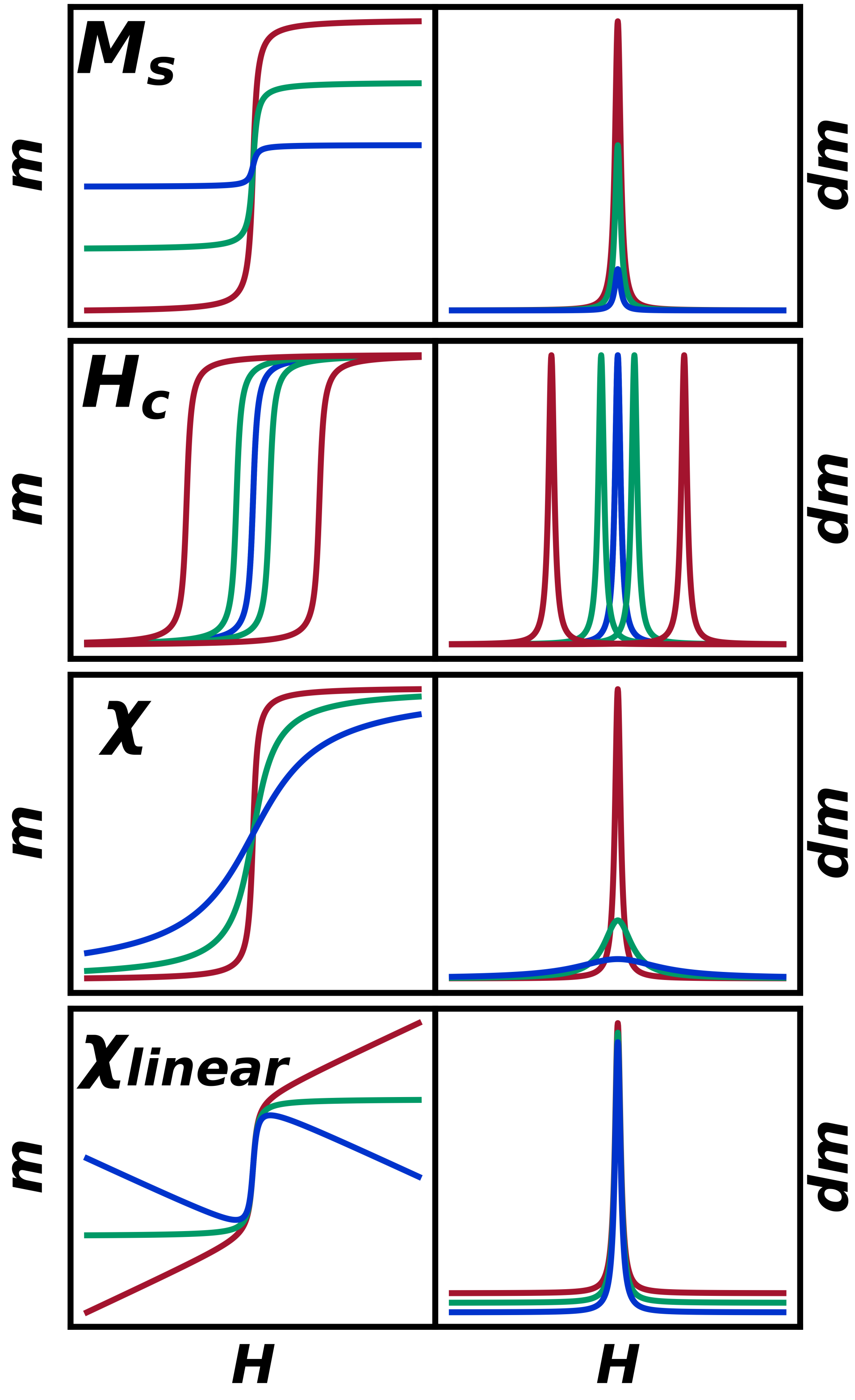

Figure S1. Magnetization curves (left) and corresponding differential magnetization curves (right) with varying parameters of M_s, H_c, χ, H_c, and χ_{linear}.

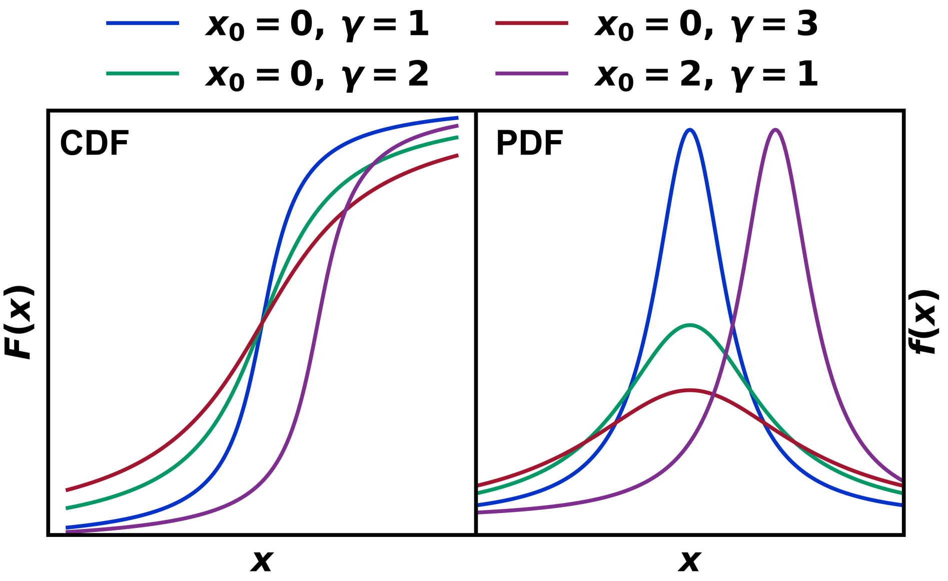

Figure S2. Cauchy distribution with varying location parameters (x_0) and scale factor (γ) in its (a) cumulative distribution function (CDF) form and (b) probability distribution function (PDF) form.

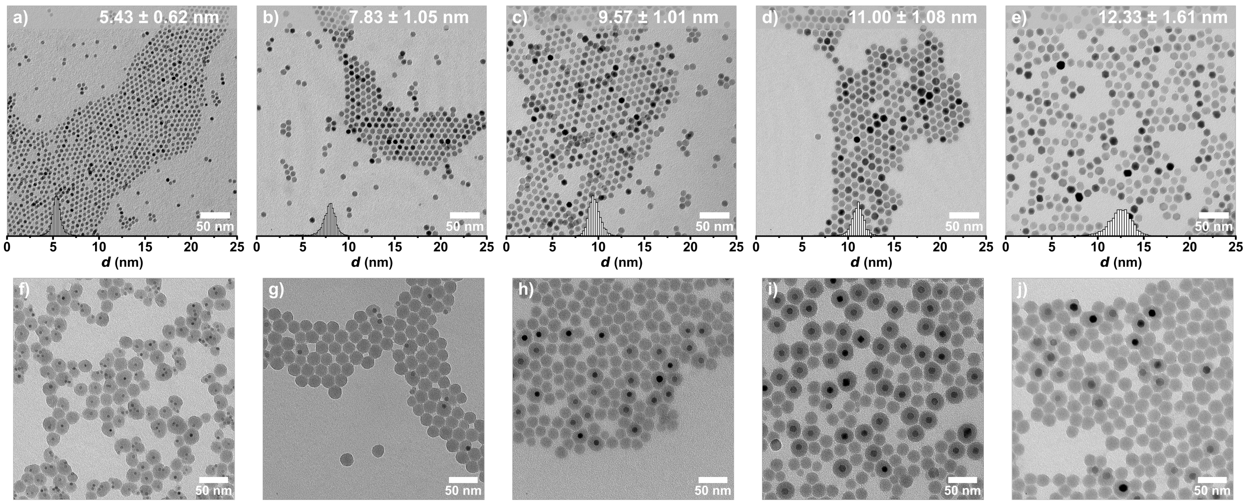

Figure S3. TEM images of (a-e) Fe_3O_4 nanoparticles and (f-j) Fe_3O_4@SiO_2 nanoparticles.

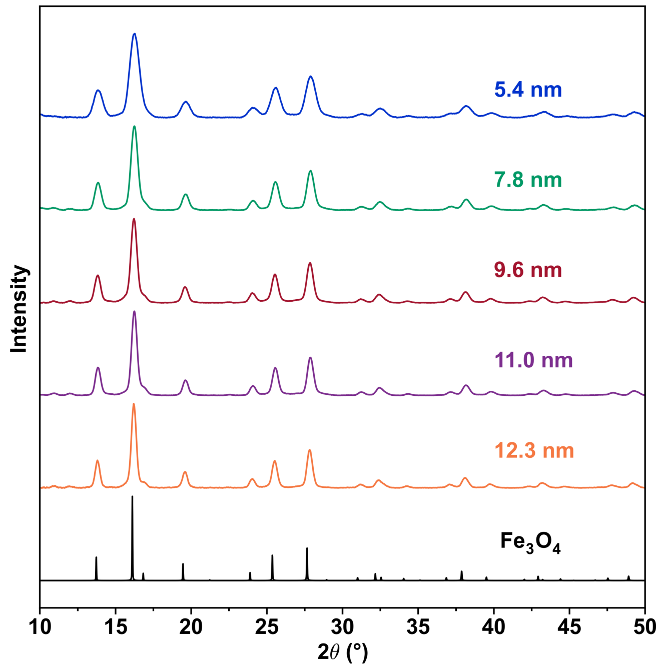

Figure S4. Powder X-ray diffraction (PXRD) patterns for the five Fe_3O_4 nanoparticle sizes, collected using a Mo Kα (λ = 0.71073 Å) source.

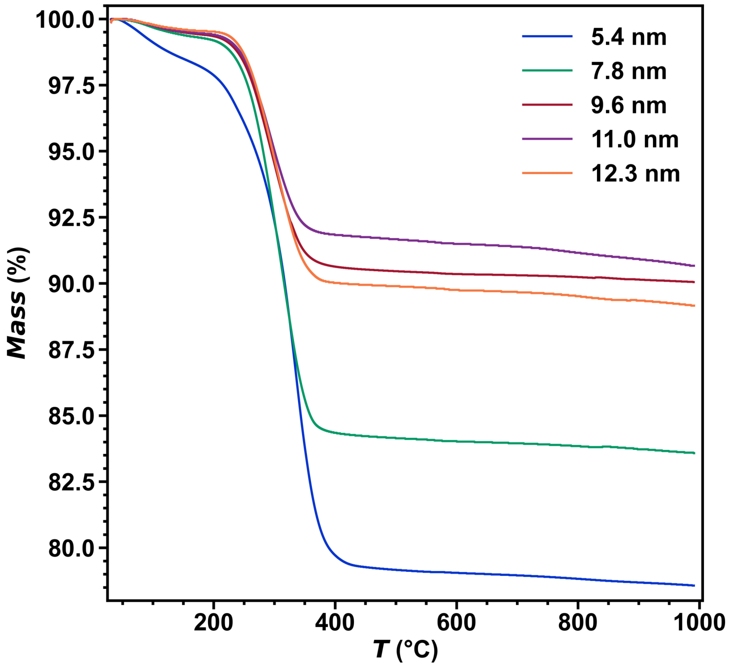

Figure S5. Thermogravimetric analysis (TGA) for the five Fe_3O_4 nanoparticle sizes, collected under air from 30 °C to 1000 °C at 10 °C/min. The Fe_3O_4 mass percentage was calculated after accounting for the 3.40% mass increase for the conversion of Fe_3O_4 to Fe_2O_3.

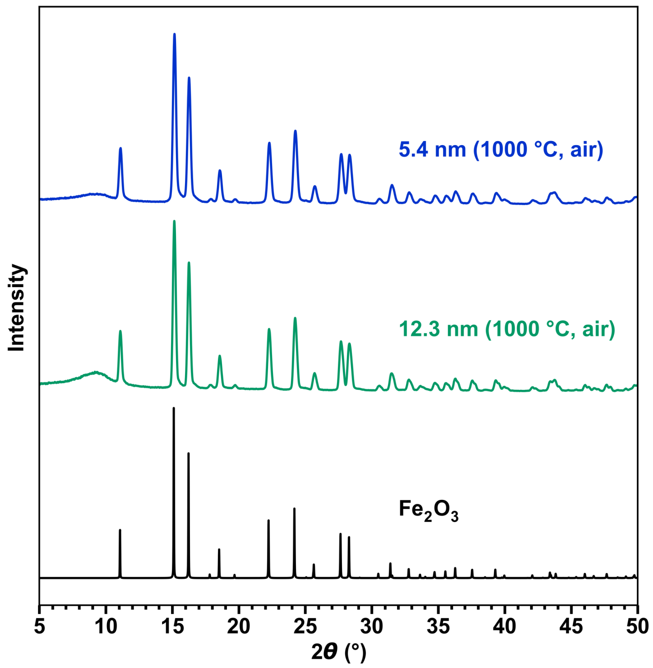

Figure S6. Powder X-ray diffraction (PXRD) patterns after TGA (Fig. S3) for the 5.4 nm and 12.3 nm samples, collected using a Mo Kα (λ = 0.71073 Å) source.

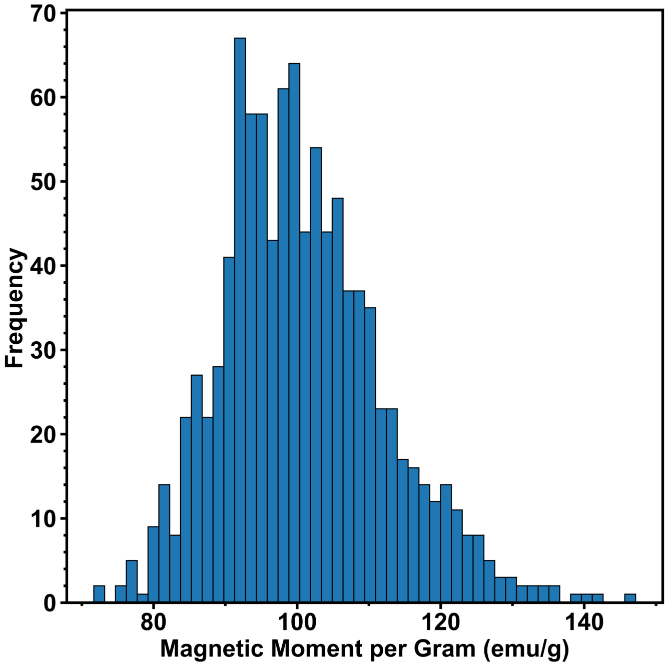

Figure S7. Histogram of magnetic moment per gram generated with 1000 samples following randomized normal distributions in both mass and magnetic moment. Typical values with a mass of 1.0 ± 0.1 mg and a magnetic moment of 0.1 ± 0.005 emu were used.

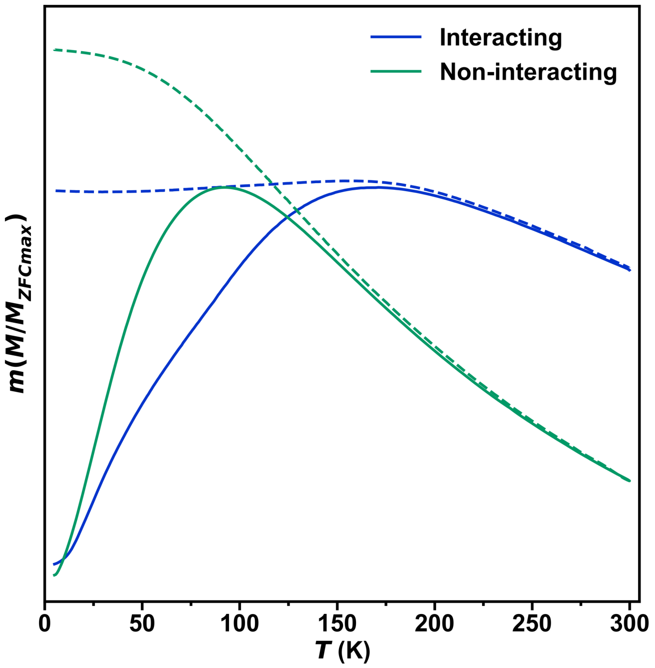

Figure S8. Plots of normalized zero-field cooled magnetization (solid) and field cooled magnetization vs. temperature (dashed) from 5-300 K for a nanoparticle system containing interparticle interactions (blue) and no interparticle interactions (green).

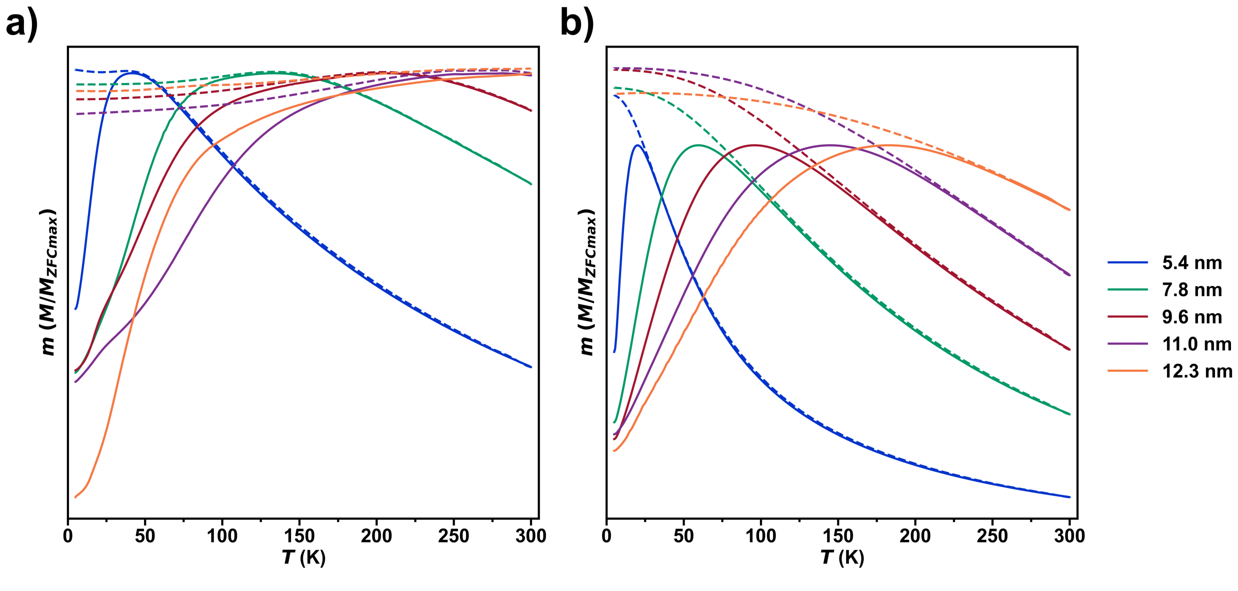

Figure S9. Plots of normalized zero-field cooled magnetization (solid) and field cooled magnetization (dashed) vs. temperature from 5-300 K under an applied field of 0.01 T for (a) Fe_3O_4 and (b) $Fe_3O_4@SiO_2$.

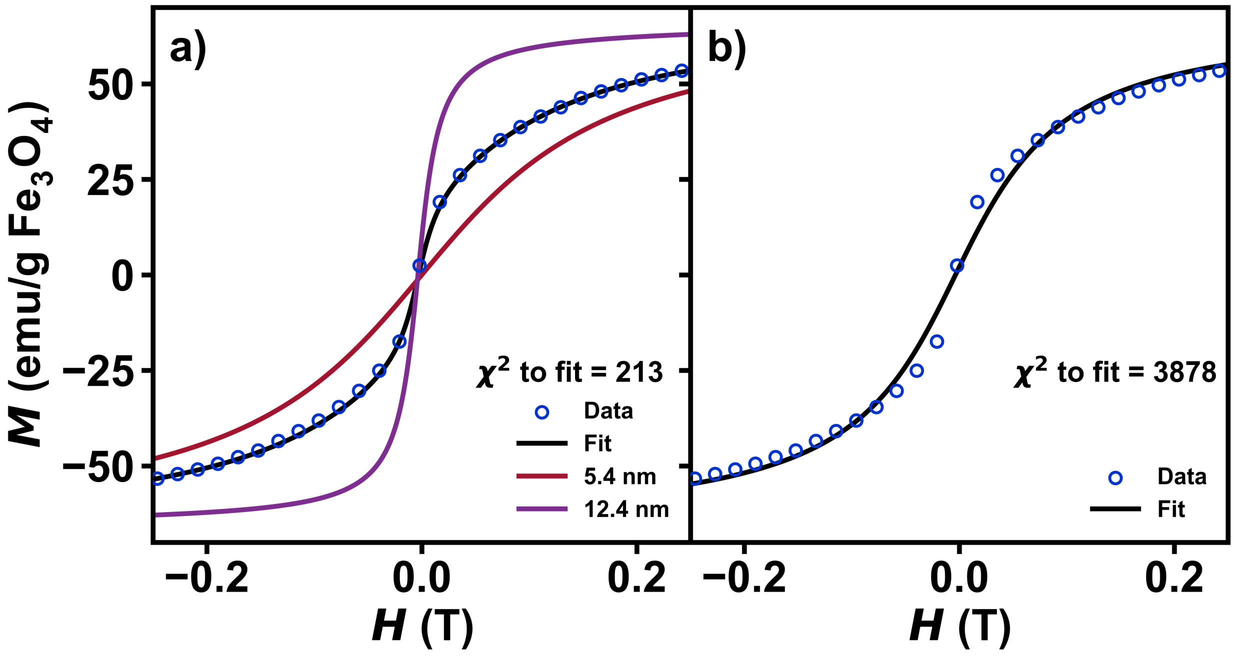

Figure S10. Magnetization curves for a physical mixture of 5.4 nm Fe_3O_4@SiO_2 and 12.3 nm Fe_3O_4@SiO_2. (a) Plot of isothermal magnetization vs. magnetic field for the physical mixture. The fit is shown in black, with contributions from 5.4 nm and 12.3 nm shown in red and purple, respectively. The fit was carried out using a sum of two unique Cauchy functions, with a relative ratio between the two, *p*, as an additional fit parameter. The γ and H_c parameters from the individual samples (Table S3) were held constant, while M_s and χ_{linear} were allowed to vary to account for mass errors. (b) Plot of isothermal magnetization vs. magnetic field for the mixture with fit to a single Cauchy function.

References

(1) Kirkpatrick, K. M.; Zhou, B. H.; Bunting, P. C.; Rinehart, J. D. Size-Tunable Magnetite Nanoparticles from Well-Defined Iron Oleate Precursors. *Chem. Mater.* **2022**, *34* (17), 8043–8053. https://doi.org/10.1021/acs.chemmater.2c02046.

(2) Ding, H. L.; Zhang, Y. X.; Wang, S.; Xu, J. M.; Xu, S. C.; Li, G. H. Fe_3O_4@SiO_2 Core/Shell Nanoparticles: The Silica Coating Regulations with a Single Core for Different Core Sizes and Shell Thicknesses. *Chem. Mater.* **2012**, *24* (23), 4572–4580. https://doi.org/10.1021/cm302828d.

(3) Dumas, R. Correcting for the Absolute Field Error Using the Pd Standard; Application Note 1500-021, Rev. B0. **2020**.

(4) Dumas, R. Using SQUID VSM Superconducting Magnets at Low Fields; Application Note 1500-011, Rev. A0. **2010**.
